# “It’s Like a Phantom Disease”: Patient Perspectives on Access to Treatment for Chagas Disease in the United States

**DOI:** 10.4269/ajtmh.17-0691

**Published:** 2018-01-29

**Authors:** Colin J. Forsyth, Salvador Hernandez, Carmen A. Flores, Mario F. Roman, J. Maribel Nieto, Grecia Marquez, Juan Sequeira, Harry Sequeira, Sheba K. Meymandi

**Affiliations:** Center of Excellence for Chagas Disease at Olive View–UCLA Medical Center, Sylmar, California

## Abstract

Chagas disease (CD) affects > 6 million people globally, including > 300,000 in the United States. Although early detection and etiological treatment prevents chronic complications from CD, < 1% of U.S. cases have been diagnosed and treated. This study explores access to etiological treatment from the perspective of patients with CD. In semi-structured interviews with 50 Latin American–born patients of the Center of Excellence for Chagas Disease at the Olive View–UCLA Medical Center, we collected demographic information and asked patients about their experiences managing the disease and accessing treatment. Patients were highly marginalized, with 63.4% living below the U.S. poverty line, 60% lacking a high school education, and only 12% with private insurance coverage. The main barriers to accessing health care for CD were lack of providers, precarious insurance coverage, low provider awareness, transportation difficulties, and limited time off. Increasing access to diagnosis and treatment will not only require a dramatic increase in provider and public education, but also development of programs which are financially, linguistically, politically, and geographically accessible to patients.

## INTRODUCTION

Chagas disease (CD), caused by the protozoan *Trypanosoma cruzi*, affects more than six million people worldwide and leads to more than 7,000 deaths annually.^1–3^ It creates a greater burden of morbidity and mortality than any other parasitic infection in the Americas.^3^ CD primarily affects marginalized populations with limited access to health care.

*Trypanosoma cruzi* is spread by blood-sucking triatomine insects, congenitally, via blood transfusion and organ transplant, and through consumption of infected food. After infection, an acute phase ensues which is either asymptomatic or easily confused with a viral illness. Subsequently, the disease enters a chronic indeterminate phase where the parasites take refuge in deep tissue, especially the heart and digestive system, to avoid the body’s immune response, remaining hidden for years. The affected person remains asymptomatic and typically unaware of the infection. In 20–30 years, 30–40% of infected individuals progress to an advanced chronic stage of the disease characterized by damage to the heart, gastrointestinal tract, and/or nervous system.^4^ Early treatment with antiparasitic drugs (benznidazole or nifurtimox) can significantly delay or halt complications from chronic CD, as well as prevent congenital transmission.^5–7^ However, < 1% of cases in Latin America and the United States, and < 10% in Europe, receive this potentially life-saving treatment.^8–11^

In the United States, 326,000–347,000 Latin American–born individuals have CD.^2^ Although the disease is also transmitted locally by native species of triatomines,^12,13^ the incidence is unknown. Importantly, Latin Americans with CD do not pose a threat of infection to the nonimmigrant population now that blood transfusions and organ transplants are fully monitored. CD is not contagious through person-to-person contact. Triatomines and *T. cruzi* are part of the natural environment in much of the United States,^14^ and autochthonous transmission has taken place since pre-Colombian times.^15^ Therefore, halting immigration flows from Latin America would never succeed in eliminating CD from the United States and would do nothing to help those who have been infected by U.S. triatomines, who are also impacted by many of the barriers described in this article.

Most of the people with CD do not realize they are infected. A host of barriers impedes diagnosis and treatment^9^: low awareness among providers,^16–18^ a lack of routine screening programs outside of blood donations, nonexistent health education campaigns, challenges in diagnosis, regulatory barriers impacting availability of benznidazole and nifurtimox,^9^ and the side effects these drugs produce.^19,20^ For Latin American immigrants, these barriers compound sociopolitical inequalities that make health care difficult to access.

Patients with *T. cruzi* infection have a range of health-care needs which presently languish in nearly total neglect. At minimum, they should receive an electrocardiogram and echocardiogram to determine cardiac involvement. Patients from South America may also require a gastrointestinal evaluation. If the patient is less than 50 years old and in the indeterminate stage, without significant evidence of cardiomyopathy, etiological treatment with benznidazole (60 days) or nifurtimox (90 days) should be offered. Before treatment, a series of laboratories, including complete blood count and renal and hepatic function, are administered. These should be repeated every 2 weeks until treatment concludes. Subsequently, annual follow-up is recommended with an electrocardiogram, echocardiogram, and serologic testing (however, seroreversion typically takes years in chronic patients).^21,22^

Prior U.S. research has examined barriers to medical care for CD from the perspective of providers, experts and policymakers,^9^ or gauged awareness of CD in Latin American immigrants.^23,24^ However, the experiences and perspectives of people living with CD in the United States are largely unexplored. This study investigates the following research questions: Who are the people with CD in the United States? How do they experience CD, confront barriers, access care, and manage their health? Answers to these questions can inform public health programs while conveying the human and social dimensions of CD.

## METHODS

The Center of Excellence for Chagas Disease (CECD) at the Olive View–UCLA Medical Center in Los Angeles is one of a handful of providers treating the disease in the United States. Founded in 2007, the CECD has screened more than 8,000 Los Angeles residents using a community-based model.^25^ In the only large-scale U.S. prevalence study outside of blood banks, the CECD found 1.24% of 4,755 Latin American–born individuals screened in Los Angeles were positive for *T. cruzi*.^26^ The CECD is housed within a safety-net facility in the San Fernando Valley on the north side of Los Angeles, the U.S. metropolitan area with the largest Latin American–born population (2.5 million). Median income for this population ($24,000) is less than half that of non-Latino whites in Los Angeles County.^27^

**Figure 1.**
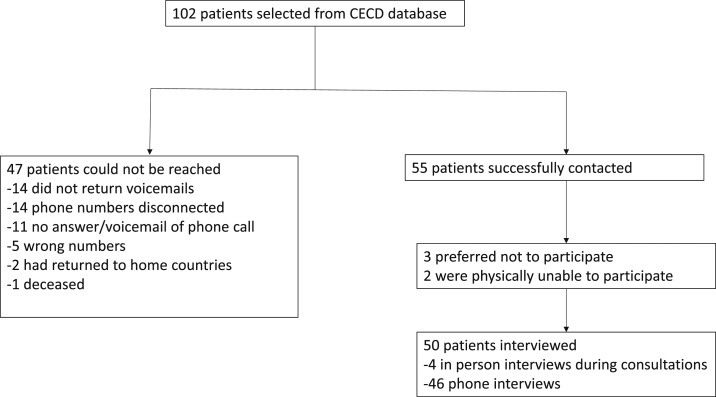
Recruitment process, Center of Excellence for Chagas Disease (CECD) patients. The process of recruitment of patients of CECD in Los Angeles for participation in the study.

A semi-structured interview questionnaire was administered to a convenience sample of 50 patients with CD who were recruited from CECD records (Figure 1). Demographic information was collected and patients were asked about their experiences with CD and the steps they took to access treatment. All participants were adults born in Latin America. Four interviews took place in person at the CECD and 46 via telephone. One interview was conducted in English and the rest in Spanish. The Institutional Review Board of the Education and Research Institute at the Olive View–UCLA Medical Center approved the study. A verbal informed consent process was used and all patient names have been changed in the article text to protect patients’ anonymity. Patients were asked for consent to record the interview, which all but one granted. Interview recordings were transcribed in Microsoft Word (Microsoft Corp., Redmond, WA), then coded for themes using qualitative analysis software (QDA Miner Lite v. 2.0, Provalis Research, Montreal, Canada). Coding involved an inductive process based on the social constructionist version of grounded theory^28^; themes emerged mainly through our analysis of the interview texts, yet we were also cognizant of prior research on barriers to CD diagnosis and treatment, as well as the historical experience of the CECD in confronting many of these barriers.

## RESULTS

### Sample characteristics.

Two-thirds of participants were female and most of them originated from El Salvador (Table 1). Participants had lived in the United States for a mean of 21.8 years; only four indicated a length of residence less than 10 years. The sample exhibits high levels of socioeconomic marginalization; participants had a mean of 8.3 years of education and only 40% had completed high school. Another 40% had not advanced beyond primary school. Of those in the workforce, the majority (25/29, 86.2%) were employed in blue collar or service occupations such as cleaning, construction, landscaping, or food service. Most patients reporting household income (26/41, 63.4%) were below the federal poverty line based on household size. Mean household income was $31,122.63 and mean household size was 3.92. The bulk of the participants relied on Medicaid. Thirteen patients already exhibited evidence of cardiomyopathy, conservatively defined as left ventricular ejection fraction lower than 45% (Table 2). Forty patients had undergone etiological treatment with benznidazole or nifurtimox, although six were unable to tolerate one or both drugs because of side effects. Ten patients had not been treated, either because of an advanced disease state (*N* = 5) or because of lack of time or patient preference.

**Table 1:** Demographic profile of Los Angeles Chagas disease patients

	*n*	%
Sex		
Male	17	34
Female	33	66
Education		
High school graduate	20	40
< High school	30	60
Country of origin		
El Salvador	28	56
Mexico	13	26
Bolivia	4	8
Guatemala	3	6
Other (Argentina, Honduras)	2	4
Occupation		
Labor/service	25	50
Homemaker	16	32
Management/professional	4	8
Retired or unemployed	5	10
Household income*		
Below federal poverty line	26	63.4
Above federal poverty line	15	36.6
Health insurance		
Medi-Cal, Medicaid, or county program	35	70
Medicare	2	4
Private	6	12
Uninsured	5	10
Not sure/unspecified	2	4

* Nine individuals were unsure/did not report income.

**Table 2:** Clinical profile, Los Angeles Chagas disease patients

	*n*	%
Disease stage		
Indeterminate	32	64
Advanced (cardiomyopathy)	13	26
No data	5	10
Treatment history*		
Received etiological treatment	40	80
Benznidazole	19	47.5
Nifurtimox	17	42.5
Benznidazole and nifurtimox	4	10
Discontinued treatment (side effects)	6	15
Untreated	10	20
Due to advancement of the disease	5	50
Other (patient declined or unable)	5	50

* Percentages shown under treated and untreated categories are percentages within those subgroups.

### Challenges in accessing health care.

To gauge potential needs and barriers, patients were asked to describe in their own words if anything had made it difficult to go the doctor in the past year. They were also asked an open-ended question: What would make it easier to go to the doctor? Table 3 lists factors patients deemed would facilitate getting medical care, including transportation, insurance, financial support, and more time off.

**Table 3:** Would anything make it easier to go to the doctor?

	*n*	%
Transportation	12	24
More insurance coverage	10	20
Financial support	10	20
More/easier time off	8	16
Less delays/wait time/bureaucracy in health-care system	7	14
Services near me	4	8
Nothing/don’t know	14	28

Responses of Los Angeles Chagas disease patients. Some respondents listed multiple factors.

#### Insurance.

Whereas some respondents had private insurance, most were covered by Medi-Cal, California’s state Medicaid program. Undocumented immigrants and certain classes of legal immigrants are barred from the Affordable Care Act and full Medicaid. However, some state programs provide limited coverage to these groups. Even undocumented immigrants may be able to quality for “emergency” Medi-Cal, a California program which provides basic coverage for pregnancy and emergency services. Eleven respondents indicated they had this type of Medi-Cal. Moreover, county programs in Los Angeles and nine other California counties provide primary care coverage to undocumented immigrants; four respondents were enrolled in such programs. Five individuals lacked insurance; two specified this was due to their immigration status. Teofilo, a 44-year-old Salvadoran, was legally in the United States working as a cook with an employment authorization card. He stated he could no longer obtain medical care for his CD, “Because I don’t have Obamacare. And after I finished my treatment the government enacted a law that people who had employment authorization could no longer get treatment… that I no longer qualified, so I stopped going to my appointments.” Even respondents with Medi-Cal expressed concern about the precariousness of their insurance coverage. “Lately I’ve had problems because they want to take away my Medi-Cal,” said Paola, 60, a cashier, “because I started working, and I’m afraid because with this Chagas, I don’t know.”

Nonetheless, insurance coverage does not necessarily facilitate treatment. Damián, 41, a Salvadoran construction worker, mentioned he had not gotten health care in the previous year, despite having coverage, “because I have to pay if I go the doctor.” Zaida, a 54-year-old housekeeper, stated: “If I know I have to pay [for an appointment] I’d rather not go,” noting that her employer-based insurance gave her “few visits” to the doctor. “Since my husband and I both work I don’t qualify for Medi-Cal. And if I had Medi-Cal I know I could keep getting treated for Chagas.”

Patients with private insurance ironically encountered additional bureaucratic delays. Because the CECD is part of a county, safety-net hospital, patients with Medi-Cal were eligible to get services. However, private insurers are often unfamiliar with CD and made patients consult specialists or subjected claims to lengthy internal review. “It takes a long time with my insurance,” said Eleana, a 33 year old from Bolivia who had insurance through her employer, “especially when I need to see [the CECD], because there is always a delay in the request.” These obstacles required patients to self-advocate with insurers, sometimes creating delays of more than a year, whereas in other cases, patients simply gave up in frustration.

#### Transportation.

Los Angeles is the largest metropolitan area by size and second largest by population in the United States. Public transportation struggles to connect the city’s far-flung neighborhoods. When asked what would facilitate going to the doctor, the most frequent response (*N* = 12) involved transportation. Several respondents lacked automobiles and some reported needing 3 hours to travel to the CECD by bus. Others depended on relatives and neighbors for rides.

Before I couldn’t go [to the doctor], because I didn’t know how to drive. I had to wait for someone to take me; I depended on someone giving me a ride as a favor. And then I didn’t have money to pay for the appointment or the ride, or sometimes for lack of time, and I’ve had to neglect other tasks so I could go to the doctor. (Renata, 36, Mexico)

Having to pay for a ride could pose a barrier. Lorinda, a 57-year-old Salvadoran, detailed significant transportation costs to go to the CECD: “When they take me and bring me back… I have to give $20 for gas and $20 to [the driver].” When asked what would help her go to the doctor, Beatriz, a 51-year-old Honduran widow, responded, “Finding a person to give me a ride, because my daughter doesn’t know how to drive and we have to go on the bus. I don’t have a car so that would help me a lot.” Some respondents specified that they did not know how to drive because they had always used public transportation in their home countries.

#### Time.

Because of challenges with transportation and waits at providers, time posed another barrier to accessing treatment. Carlota, a 64 year old from Guatemala who worked cleaning houses, explained: “if you live a long distance away, you have to take the whole day and you have to have money to buy water, food, while you’re waiting for your appointment.” Respondents were particularly concerned that the time needed to travel to the clinic and wait for appointments conflicted with work schedules. “Sometimes they don’t want to give you time off at work,” said Marisol, 36, who worked in retail. Paola said “The problem is I have no time, sometimes I just have one day off… I lose the whole day.” Omar affirmed, “There’s never enough time” for appointments, “since I’m always working.”

Moreover, family members may have limited time off available to care for patients undergoing chronic complications from CD.

When I was sick my husband lost two days of work to take me to the hospital, and also my son was with me for three months after treatment, taking 3 days of vacation plus his two days off so he could be with me five days a week. (Sara, 60, El Salvador)

For patients and relatives working in service or construction jobs, “time off” typically meant time without pay, imposing additional economic hardships on families.

### Awareness of CD.

#### Patient awareness.

Chagas disease is seldom publicized, either in patients’ home countries or the United States. Many had never heard of CD until their diagnosis, leading to surprise and confusion. “I was confused because… I had never heard of the disease, I didn’t know anything about it,” said Sofia, 43, who came to the United States from El Salvador as a child. For most of the people in the indeterminate phase of the disease, like Eleana, the diagnosis is confusing because of the lack of symptoms. “I don’t feel anything. They told me I have Chagas but I don’t know what it is or how it feels.” Other patients express surprise that the disease was not detected during their regular physical examinations.

When patients had heard of CD, it was typically because a family member had been impacted. Five participants mentioned CD had caused the death of a close relative. “My brother died and they detected CD, and there was nothing they could do for him,” said Lorinda. “It really affected me,” said Victoria, a 43-year-old Salvadoran who worked cleaning houses, after being diagnosed. “Because in my family people died of Chagas disease, so I knew a little from their stories. So it really affected me, it scared me.”

Other participants point to a lack of discussion of CD in society and the media.

It’s a fatal disease, and yet you don’t hear anything about it, it’s like a phantom disease that is killing people but nobody knows it exists, until they tell you, you have it. You always hear about diabetes, cancer, but [Chagas] disease is something that’s never heard anywhere, not even in the media. (Sara, 60, El Salvador).

On TV you don’t hear anything about it, and all of us Hispanics watch TV and listen to the radio, and yet I never heard anything about Chagas… And since there is no discussion of this topic, many people don’t even know Chagas exists. (Sandra, 54, Mexico)

These respondents were left wondering why this potentially fatal disease was so absent from public discourse.

#### Awareness in the medical community.

Low awareness of CD extends to the medical community, creating a major barrier for patients seeking treatment. Because CD is not routinely screened in primary care, people usually find out they are infected after giving blood. By 2007, most states began screening blood donations for *T. cruzi* to prevent infections through transfusions. Sofia was one of the first cases detected after she donated blood in 1997, but was unable to initiate treatment until 2008:

I thought I had a doctor, a clinic who could help me, but Chagas taught me this is not always so. Because nobody, I mean nobody knew what Chagas disease is. My doctor didn’t know, and he sent me to a specialist, and every week I went to a different doctor, but nobody knew what to do with me. And after a year I gave up.

When donors test positive for *T. cruzi*, they receive notification from the Red Cross through a letter or phone call and are advised to consult their doctor. Several respondents described struggles in obtaining help from the medical system after their blood tests positive because providers are unaware of what CD is:

They explained to me I had been bitten by a chinche [kissing bug]… and not to worry too much, to talk to my doctor, but I started to search and nobody could help me because they didn’t know what the disease was. (Denver, 42, El Salvador)

Victoria was diagnosed after giving blood in El Salvador and began getting treatment from an infectious disease specialist, riding from her village to the clinic in the back of a truck, but had to stop because she could not afford it. Later, she moved to the United States “with the hope that here I would be able to get treatment.” Unable to qualify for Medicaid, she went to a private clinic, but the doctor misunderstood when Victoria attempted to explain she had CD and instead tested her for Lyme disease. Subsequently, Victoria despaired of finding treatment until volunteers from the CECD came to a health fair at her church.

When Omar sought help from his doctor after being diagnosed with CD, he was met with confusion and incomprehension. “I had to show the doctor on Google what Chagas is,” he recalled. These experiences underscore how difficult it is for patients to connect with providers who know how to treat CD.

### Language.

The bulk of respondents (*N* = 30) identified language as the most difficult aspect of adjusting to the United States. Although the CECD has bilingual staff, patients reported language differences could complicate their efforts to seek care from other providers. Jimena preferred to return to Mexico when she needed attention for the heart condition caused by her CD. “I get care in Mexico because I can communicate with the doctors. Since I don’t speak English, here it’s very difficult…” Because of this language gap, when she suffered a heart attack, she preferred to have her husband drive her across the border to a hospital in Mexico, a 3-hour trip, rather than calling an ambulance in Los Angeles.

Communication barriers with providers are shaped not only by language, but by class and educational differences, which could make it difficult for patients to understand clinical terminology used by health-care providers. “Since I don’t know how to read, sometimes I don’t understand the words [the doctors] tell me,” said Marta, 65. Education and language barriers make it extremely challenging for patients to explain CD to unfamiliar providers or insurers.

### Solutions for CD.

Patients were asked what they would do to help people with CD if they were the President of the United States (Table 4). Patients’ solutions focused on raising awareness, improving medications, expanding screening, increasing the number of providers offering treatment, and making treatment more accessible. Their solutions reflect the barriers they have had to overcome to find treatment.

**Table 4:** If you were the U.S. President, how would you help people with Chagas disease?

Solution	*N* (%)	In the patients’ words
Provide more information	13 (26)	There is not a lot of awareness. I think every health center should give talks about this disease to train the nurses and everyone else because they don’t have knowledge about it. (Roberto)
Find new/better medicines	13 (26)	In my case they can’t give it to me because it’s very strong and if the medicine were a little milder I could take it. (Jorge)
Increase screening	9 (18)	If I were President, I would include it in annual physicals or provide the test for free. I would find a way to provide free Chagas testing in more places. (Renata)
More facilities, providers offering treatment	8 (16)	Make treatment more accessible. Thank God we have insurance and were able to go to this clinic, but maybe if there were more clinics and better informed doctors it would be easier. Because there’s just one clinic, so far away and nobody else knows about this disease. (Eleana)
Provide free or more accessible treatment	7 (14)	I would provide free services for all people with the disease, and explain to them what the disease is and how they can survive. (Flor)

## DISCUSSION

Other research on barriers to treatment of CD has taken a system-level perspective. Manne-Goehler et al.^9^ identified multiple barriers, including a complicated diagnostic process, limited follow-up of positive blood donors, lack of organized systems of care and financing for the disease, low provider awareness, lack of Food and Drug Administration (FDA) approval of medications, and limited investment in research and education. The study noted that only 422 courses of treatment had been released by the Centers for Disease Control and Prevention (CDC) from 2007 to 2013, which would not even cover 0.2% of the most conservative estimate of expected cases nationally. Similar systemic barriers were identified in studies of access to treatment in Colombia^10^ and Mexico.^8^ Our research supports Manne-Goehler et al.’s findings for the United States and underscores the importance of insurance coverage, follow-up of blood donors, health education, and provider awareness. Moreover, patients identify language, transportation, time off from work, and side effects from treatment as additional barriers.

In our study, increasing provider awareness was a major need emphasized by patients. Other U.S. research has investigated low awareness of CD. In a prior CECD study, 86% of Latin American–born individuals in Los Angeles had never heard of CD.^24^ Stimpert and Montgomery^16^ analyzed survey data of 1,142 U.S. physicians: 47% of obstetricians/gynecologists and 23% of cardiologists had never heard of CD. Even in physicians who were aware of CD’s existence, a large segment did not feel confident that their knowledge of the disease was up to date.^16^ Other studies indicate physicians do not consider CD in their diagnoses, even when patients are from endemic countries.^17,18^

The FDA recently approved benznidazole, the first-line medication for CD, for use in children 2–12 years old. This removes a major bureaucratic barrier for physicians, but prescriptions for patients outside this age range will be considered “off-label.” With most U.S. Chagas patients being older than 12, it is imperative that the indication for the drug be expanded as broadly as possible to facilitate physicians’ use of benznidazole for treatment.

Many barriers impeding patients’ access are rooted in sociopolitical inequalities impacting Latin American immigrants. A study predicts that half the projected 2.7–3.4 million uninsured in California in 2019 will be undocumented immigrants, despite state programs designed to cover this population.^29^ Bureaucratic complexities and fear of deportation limit enrollment. Even after passage of the Affordable Care Act, 47% of Mexican immigrants still lack insurance coverage (compared with 9% of the U.S.-born population).^30^ An estimated 30% of the Latin American–born population with CD is undocumented,^2^ and research suggests this has a significant impact on treatment seeking for CD.^9,23,31^ In our study, this barrier was partially mitigated by Medi-Cal emergency coverage and other local programs. The situation is apt to be bleaker in states which have refused federal funds to expand Medicaid, including Texas and Florida, which rank second and third nationally in terms of CD burden.^2^

There is a strong link between socioeconomic conditions and CD.^32^ Jackson et al.^33^ examined socioeconomic conditions among 137 immigrants with CD in Switzerland. The majority (89.1%) lived below the Swiss poverty line, compared with 63.4% in our study living below the U.S. poverty line. Socioeconomic conditions may play an important role in disease progression. A study in Argentina of 801 patients with CD found having health insurance and more years of education were protective factors against progression to chronic cardiomyopathy.^34^ Similarly, we found that education and language differences posed a significant barrier, compounded by low provider awareness. Lack of insurance posed another key barrier, yet bureaucratic delays with private insurers also impeded treatment. Furthermore, some patients could not afford transportation or had no time off benefits, making it extremely difficult to go to appointments. Geographic distance of providers offering treatment was identified as a major barrier in studies in Latin America as well.^10,35^

Our study has several limitations. Because the sample consisted of patients who were able to obtain diagnosis at the CECD, they are not representative of the > 99% of CD patients who remain undiagnosed. Furthermore, 40 respondents received etiological treatment, which is even rarer. We use a convenience sample; patients in the study are more likely to live near the CECD and to be affiliated with churches and other organizations connected to the CECD’s outreach program. Although we attempted to reach 102 current or former patients, we could only connect with 55. The population served by the CECD may be harder to contact because of high mobility, restrictions on time due to work, difficulties with immigration status, or apprehension about interacting with official institutions. The patients we were not able to contact may actually face more severe barriers to accessing health care. Most patients’ information about CD came from their interactions with the CECD, which could have influenced their responses.

With < 1% of U.S. patients diagnosed, there is a need for widespread screening. However, diagnosed patients will struggle to find treatment until there is a concerted effort to educate health-care personnel on CD. Moreover, both Medicaid and private insurers should cover treatment in a straightforward manner. Ideally, treatment of CD should be available in primary care facilities near to where patients live. Information on CD should be available in straightforward terms in language that is familiar to patients, and there is a critical need for increased public awareness campaigns.

United States CD programs cannot ignore the constellation of political, economic, and cultural factors which severely limit this group’s access to the health-care system. Our study highlights the importance of patients’ transportation needs, employment situation, family/social support systems, and linguistic preferences for programs designed to increase access to CD health care. Reaching the 99% of U.S. patients who presently do not receive diagnosis and treatment of CD will require a strong commitment from government, public health entities, providers, and insurers, based on the premise that access to care for a life-threatening disease should be a fundamental human right.
